# Whole-genome sequencing of two clinical *Staphylococcus haemolyticus* isolates highlights antibiotic resistance genes and prophage elements

**DOI:** 10.1128/mra.00773-25

**Published:** 2026-01-28

**Authors:** Fariza Shams, Syeda Naushin Tabassum, Aura Rahman, Nayma Haque Tonny, Pronoy Debnath, Asifa Siddika Badhon, Abdus Sadique, Jahidul Alam, Maqsud Hossain

**Affiliations:** 1 Department of Biochemistry and Microbiology, School of Health and Life Sciences, North South University 54495, Dhaka, Bangladesh; 2 NSU Genome Research Institute (NGRI), North South University, Dhaka, Bangladesh; Fluxus Inc., Sunnyvale, California, USA

**Keywords:** *Staphylococcus haemolyticus*, antibiotic resistance, virulence gene, prophage, whole-genome sequencing

## Abstract

*Staphylococcus haemolyticus* is an emerging, multidrug-resistant pathogen associated with healthcare infections. We report the whole-genome sequences of two human isolates, NGCE 403 (2.6 Mb, 33% GC content, 2,533 genes) and FSCC13 (2.6 Mb, 32.5% GC content, 2,586 genes), which provide insights into their virulence and resistance mechanisms.

## ANNOUNCEMENT

*Staphylococcus haemolyticus*, a coagulase-negative staphylococcal species, is frequently associated with bloodstream and device-related infections and is characterized by antibiotic resistance, biofilm formation, and adaptation to a variety of host-associated niches, complicating therapy and increasing infection risk (1, 2).

*S. haemolyticus* strains NGCE403 (from stool) and FSCC13 (from wound) were isolated from patients at a hospital in Dhaka (23.8041° N, 90.4152° E). The stool sample, homogenized in sterile PBS, and the wound swab were streaked onto mannitol salt agar and incubated at 37°C for 18–24 h. Pink colonies were subcultured on TSA (Thermo Scientific) and presumptively identified by Gram staining and catalase, oxidase, coagulase, urease, and novobiocin susceptibility tests (3). Isolates grown in tryptic soy broth at 37°C for 18-24 h were used for DNA extraction with the DNeasy Blood and Tissue Kit (QIAGEN). Nanopore sequencing libraries were prepared using the Rapid PCR Barcoding Kit (SQK-RPB004, Oxford Nanopore Technologies, UK) and sequenced using a FLO-MIN106 (R9.4.1) flow cell on the MinION Mk1C platform. Basecalling was performed using Super Accuracy mode in Guppy (v.5.0.11); trimming was performed using Porechop (v.0.2.3) (4); and filtration was performed using Filtlong (v.0.2.1) (5). Long-read assembly was performed using Flye (v.2.9) (6). Default parameters were used except where otherwise noted. Genome annotation was performed using the Prokaryotic Genome Annotation Pipeline (v.6.5) (7). Genome assemblies were analyzed with QUAST (v.5.3.0) (8), and read counts, N50, and coverage depth were calculated from raw FASTQ files using SeqKit (9) and custom Python scripts. Sequencing details for FSCC13 and NGCE403 are listed in Table 1. The draft genome of *S. haemolyticus* NGCE 403 contains 2,533 genes, including 2,109 protein-coding genes and 80 RNA genes (60 tRNAs), whereas the strain FSCC13 contains 2,586 genes, including 2,163 protein-coding genes and 79 RNA genes (59 tRNAs). By MLST 2.0 (10), the strains NGCE 403 and FSCC 13 were identified as ST 3 and ST 40, respectively. ResFinder (v.4.6.0) (11) identified aminoglycoside resistance genes, including *aac(6′)-aph(2″)*, *APH(3′)-IIIa*, and *ant(6)-Ia*, alongside *mecA* for beta-lactam resistance in FSCC13. NGCE403 contained *mecA*, *blaZ*, and macrolide resistance genes *msrA* and *mphC*. Both strains shared genes such as vanY, *vanT*, *norC*, and *sdrM*, which confer resistance to glycopeptides, fluoroquinolones, and disinfectants. Mobile Element Finder (v.1.0.3) (12) detected one plasmid in NGCE403 and two in FSCC13, carrying resistance genes for erythromycin (*ermC*, *mphC*, and *msrA*), penicillin (*blaZ*), and chlorhexidine (*qacA*). Among the virulence factors, capsular polysaccharide (capA family protein), *SasC* (cell-cell adhesion), *atl* (autolysin), *lip* (lipase), *nuc* (nuclease), and *cylR2* (cytolysin) were predicted in both strains. Strain FSCC13 uniquely harbored *clfA* (clumping factor), while strain NGCE403 uniquely contained fibrinogen-binding protein *sdrC*. PHASTEST (13) identified one prophage in NGCE403, which contains structural genes, a repressor, and integrase, suggesting potential virion production. FSCC13 has two prophages: one similar to the one in NGCE403 and another, likely cryptic, lacking structural genes. This genomic resource will enhance our understanding of lifestyle, immunotherapeutic, and chemotherapeutic approaches to control *S. haemolyticus*.

**TABLE 1:** Genome assembly and strain information for the two *S*. *haemolyticus* strains

Strain	Total reads	Read N50 (bp)	Assembly length (bp)	No. of contigs	Assembly N50 (bp)	GC (%)	Genome fraction (%)	Duplication ratio	Coverage (×, assembly)
FSCC13	105,245	4,729	2,594,099	8	610,788	32.5	89.022	1.011	≈161×
NGCE403	95,393	4,961	2,551,171	2	2,520,515	33	87.859	1.010	≈170×

## Data Availability

The genome sequences of *S. haemolyticus* strain FSCC 13 and NGCE 403 have been deposited at DDBJ/ENA/GenBank under accession numbers JBNFUP000000000 and JBNFUQ000000000, respectively. The BioProject and BioSample accession numbers of *S. haemolyticus* strain FSCC13 are PRJNA1253103 and SAMN48065002, respectively. The BioProject and BioSample accession numbers of *S. haemolyticus* strain NGCE403 are PRJNA1253111 and SAMN48065039, respectively. Raw sequencing reads of *S. haemolyticus* strain FSCC13 and NGCE403 are available in the NCBI Sequence Read Archive at https://www.ncbi.nlm.nih.gov/sra/PRJNA1253103 and https://www.ncbi.nlm.nih.gov/sra/PRJNA1253111, respectively.
